# Experimental Study of Utilizing Recycled Fine Aggregate for the Preparation of High Ductility Cementitious Composites

**DOI:** 10.3390/ma13030679

**Published:** 2020-02-03

**Authors:** Dan Ying Gao, Mingyan Lv, Lin Yang, Jiyu Tang, Gang Chen, Yang Meng

**Affiliations:** 1 School of Civil Engineering, Zhengzhou University, Zhengzhou 450001, China; gdy@zzu.edu.cn (D.Y.G.); tjy74@zzu.edu.cn (J.T.); m137953186@163.com (Y.M.); 2 School of Water Conservancy Engineering, Zhengzhou University, Zhengzhou 450001, China; yanglin06142@zzu.edu.cn; 3 School of Civil Engineering, Henan University of Engineering, Zhengzhou 451191, China; gchen@haue.edu.cn

**Keywords:** cementitious composites, mechanical properties, microstructure, fiber, recycled fine aggregate, ductility

## Abstract

Waste concrete was recycled and crushed into fine aggregate to prepare a high ductility cementitious composite (HDCC) in this study, for helping dispose the massive amount of construction waste and for reserving natural resources. Firstly, the features of recycled fine aggregate (RFA) were analyzed in detail and compared with natural fine aggregate (NFA). After that, the mechanical properties, including compression, flexure, bending and tension, and the microstructure of high ductility cementitious composite (HDCC) prepared with RFA were systematically investigated and compared with that of HDCC prepared with NFA. The results show that, since RFA has a higher water absorption rate and contains 4.86 times as much crush dust as NFA, HDCC with RFA forms a denser matrix and a higher bond between fiber and matrix than HDCC with NFA. Thus, HDCC with RFA has higher compressive, flexural, bending and tensile strength. Meanwhile, the higher bond between the fiber and matrix of HDCC with RFA and the finer particle sizes of RFA can greatly promote the development of multiple cracking. As a result, HDCC with RFA exhibits more remarkable stain hardening, and presents 182.73% higher peak deflection in bending and 183.33% higher peak strain in tension than HDCC with NFA. Finally, with the consideration of fiber volume fraction, the prediction models for the peak strengths of HDCC with RFA were proposed. The prediction results show a good agreement with the test results.

## 1. Introduction

Construction and demolition debris contribute a considerable fraction of solid waste, wherein the waste concrete constitutes the largest component with a percentage of about 70% [1]. Most of the construction waste is released in open air or dumped in landfills because of the high disposal costs, thereby causing a scarcity of cultivated lands and severe pollution in the atmosphere, aquifer and soil [2,3,4,5]. In addition, as the most important building material, millions of tons of concrete are produced worldwide each year. The raw materials, such as aggregates, which occupy about 60–75% of total concrete [6], are also consumed in large quantities [4,7]. As an efficient way to reduce damage to the environment and to save non-renewable resources, recycling and crushing the construction waste into recycled aggregate for concrete has attracted much attention [2,8,9,10,11,12]. Recycled coarse aggregate has been studied and applied to roadway construction, concrete pavement and other civil engineering works recently [4,13,14,15,16]. A large amount of fine particles with a maximum size of 0.5 mm has been produced [17] during the production of recycled coarse aggregate. These fine particles called recycled fine aggregate (RFA) have also been investigated a lot. Yang et al. [18] found that the performance of RFA containing crush dust is a significant improvement over traditional RFA with a lower water demand and higher strength of mortar. Lederer et al. [19] reported that the use of crush dust can form a good particle gradation with cement, fly ash and RFA and having a good filling effect, which then increases the compressive strength of mortar. Nili et al. [20] reported that concrete with 50% RFA replacement has reduced compressive strength, tensile strength, and energy absorption. Liang et al. [21] found that the compressive strength and elastic modulus of concrete containing both recycled coarse aggregate and RFA is lower than that of concrete containing natural coarse aggregate and RFA after enduring high temperatures. Evangelista and de Brito [22] demonstrated that the use of RFA up to 30% shows little influence on the properties of concrete.

On the other hand, as conventional concrete hardly satisfies the needs of some high-rise and large-span structures, high ductility cementitious composite (HDCC), which is characterized by strain hardening and multiple cracking, has been developed. HDCC exhibits higher strength, greater strain capacity of 3–5%, lower elastic modulus and more extreme energy absorption ability [23,24,25,26,27,28,29] compared with concrete, and has been widely applied in engineering. Furthermore, HDCC has good durability including high freezing-thawing resistance [30,31] and low water and ion permeability [32,33], thereby making it very suitable for structures in harsh environments. Sand, one of the raw materials of HDCC, is almost exhausted so using RFA as an alternative to sand can effectively alleviate the scarcity of natural resources and expand the application of waste concrete. Li and Yang [34] utilized recycled concrete fines, which was treated according to a modified Fuller’s curve, in order to replace microsilica sand for preparing engineering cementitious composites. The results prove that ECC with recycled concrete fines features a decent tensile strain capacity of several percent. Zhang et al. [35] investigated the flexural and compressive strength of ultra-high performance concrete prepared with RFA and found that the strength decreases as more RFA is introduced but is enhanced by the autoclaved curing. Yu et al. [36] added recycled fine powder to replace cement to prepare ultra high performance-engineering cementitious composites and demonstrated that recycled fine powder has an accelerating effect on the hydration of the matrix and can significantly reduce the autogenous shrinkage of UHP-ECC. However, according to the Fuller’s curve treatment, the fine particles from crushed waste concrete need to be separated by multiple screens and then remixed by specified weight. Meanwhile, meeting Fuller’s curve requires a higher proportion of crush dust, which means that more waste concrete needs to be crushed. In addition, the behavior under bending and tension of HDCC prepared with 100% RFA has not been studied in detail.

In this study, RFA was obtained by sieving the crushed waste concrete particles with only one screen of 1.18 mm. In other words, RFA with a maximum particle size of 1.18 mm and original particle size distribution was employed as the substitute of natural fine aggregate (NFA) in the preparation of HDCC. We expect to achieve two goals in this way, one is reducing the steps and dust pollution in the raw materials process, and the other is that HDCC with great bending and tensile behaviors can be prepared. The features of RFA were analyzed in detail and compared with NFA. Then, the mechanical properties of HDCC prepared with RFA (named R-HDCC in this study), including compression, flexure, bending and axial tension, were systematically investigated and compared with those of HDCC prepared with NFA (named N-HDCC in this study). The microstructure investigations of two kinds of aggregates and HDCCs were conducted to provide the arguments for mechanical analysis. Moreover, with consideration of fiber volume fraction, the prediction models for the peak strengths of R-HDCC were put forward, providing the basis for preliminary design and application.

## 2. Experimental Program

### 2.1. Materials

Ordinary Portland cement 42.5R and GradeⅠFly ash obtained from Hejin power plant (Shanxi province, China) were used in this work. Their chemical compositions are listed in Table 1. The manufacturing process of RFA includes four steps: (1) collect the waste concrete, (2) crush the waste concrete using a jaw crusher, (3) further crush the waste concrete using a hammer crusher and (4) sieve the crushed particles to a maximum size of 1.18 mm. River sand with a maximum size of 1.18 mm was used as NFA. The physical properties of polyvinyl alcohol (PVA) fiber are shown in Table 2. Polycarboxylate water reducer produced by the Subote Company was used to control the workability of mixtures.

### 2.2. Mix Proportion and Preparation of Specimens

**Figure 1:**
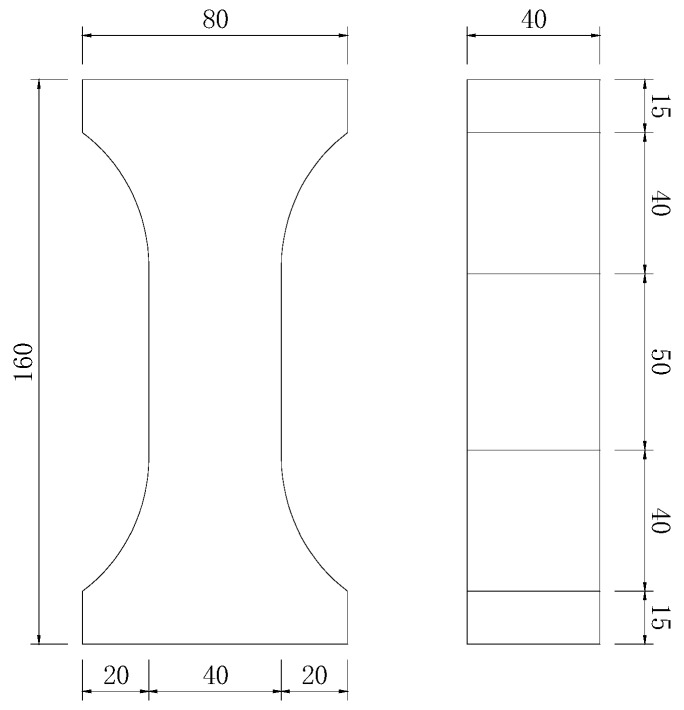
The specific dimension of dog bone specimen (dimension in mm).

The mix proportions are shown in Table 3. Ten groups of prism specimens (25R00-25N20) with the dimension of 40 mm × 40 mm × 160 mm (Width × Height × Length) were prepared to conduct the three-point flexural test and compressive test. Ten groups of slab specimens (25R00-25N20) with dimensions of 400 mm × 100 mm × 15 mm (Length × Width × Height) were prepared to carry out the four-point bending test. Ten groups of dog bone specimens (45R00-45N20) were prepared for the axial tensile test. The specific dimension of the dog bone specimens is shown in Figure 1. All specimens were prepared by the following procedure: solid raw materials including cement, aggregate and fly ash were added into the mixer together and stirred slowly for about 2 min. After that, the liquid including water and the water reducer was slowly added into the mixer and continuously stirred. When the fresh mortar was uniform, the fibers were added and mixed for 2 min, and then stirred with a high speed to increase the dispersion of fibers. Finally, the mixtures were poured into the steel molds and then vibrated on a vibrating table for 30 s. The specimens were cured for 24 h in the standard curing chamber (20 °C, RH ≥ 98%) before demolding. After demolding, all the specimens were cured in water (20 °C) until 28 days.

### 2.3. Test Method

#### 2.3.1. Flexural and Compressive Strength Test

**Figure 2:**
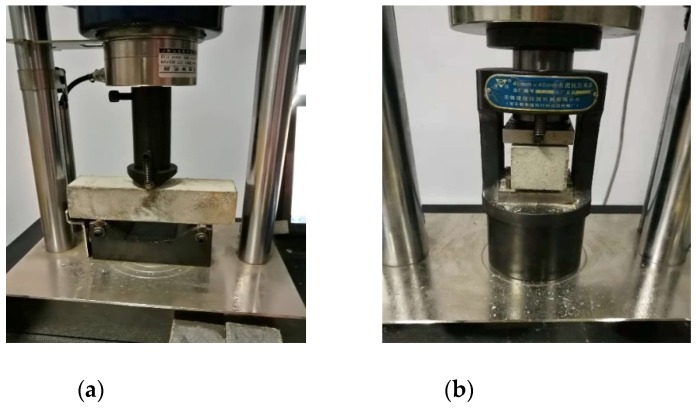
Test photos: (**a**) flexural test and (**b**) compressive test.

The three-point flexural test and compressive test were conducted on a universal testing machine (Wuxi Construction Instrument Manufacturing, Wuxi, China) of 300 kN capacity according to Chinese Standard GB/T17671-1999, as shown in Figure 2. First, the prism specimen with the span length of 100 mm was loaded in the middle span to measure the flexural strength (Figure 2a). The loading rate was 50 N/s. Afterwards, the two parts of each fractured prism specimen were placed on the test setup with an area of 40 mm × 40 mm (Figure 2b) to test compressive strength at a loading rate of 2.4 kN/s. The average flexural strength and compressive strength were determined by the three samples of each group.

#### 2.3.2. Four-Point Bending Test

**Figure 3:**
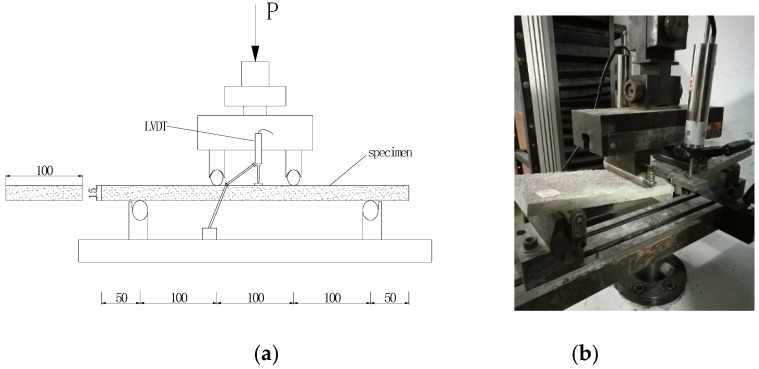
Four-point bending test: (**a**) Schematic representation (dimension in mm) and (**b**) Photo of test.

The four-point bending test was conducted on an electronic universal testing machine (SANS, MTS Industrial systems (China), Shenzhen City, China) of 50 kN capacity. The span of 300 mm was equally divided into three parts. The loading was controlled by displacement with a rate of 0.1 mm/min. Two linear variable displacement transducers (LVDTs) were mounted at the midspan to measure deflection (Figure 3). The readings of load and LVDTs were collected by data logger once per second.

According to ASTM C1018 and ASTM C78 [37,38], the load is transformed into stress by Equation (1). The point in the stress-deflection curve, where the curve begins to become nonlinear, is recorded as the first crack point, and the stress and deflection corresponding to it are defined as first crack stress *σ_bc_* and first crack deflection *δ_bc_*, respectively. The maximum bending stress in the curve is defined as peak stress *σ_bp_*, and the deflection corresponding to it is defined as peak deflection *δ_bp_*. The maximum deflection is defined as ultimate deflection *δ_bu_*. The area under the whole load-deflection curve is defined as bending fracture energy *G_b_*. The test results are the average of three samples.

(1) $$\sigma =\frac{PL}{bd^{2}},$$

where: *σ* is the bending stress, *P* is the applied load, *L* is the span length, *b* is the width of the specimen and *d* is the depth of the specimen.

#### 2.3.3. Axial Tensile Test

**Figure 4:**
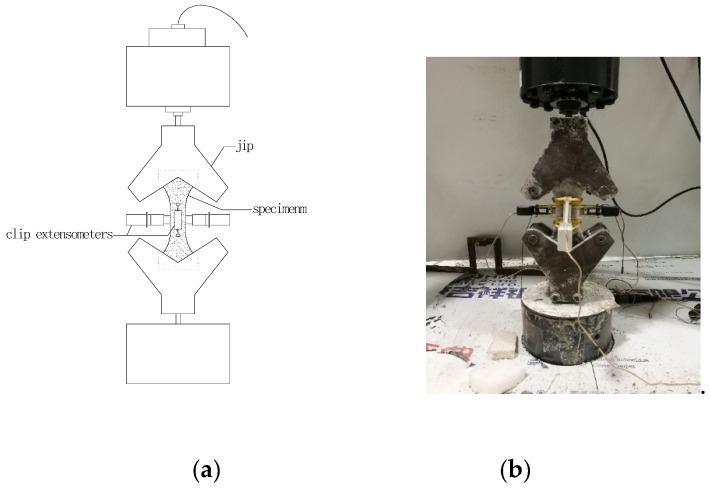
Axial tensile test: (**a**) Schematic representation and (**b**) Photo of test.

The axial tensile test was conducted on the MTS universal testing machine with a loading rate of 0.001 mm/s. Four clip extensometers were mounted at the middle segment of the specimen to measure the longitudinal and transverse elongation (Figure 4). The load and deformation were automatically recorded by MTS. The results are the average of valid samples for each series. Only the results of specimens failed in the gauge length and without eccentricity were employed. In this case, at least two results should be valid and the deviation should be less than 30% for each series; otherwise, the test of this series was repeated.

The load and deformation are transformed into stress and strain by Equations (2)–(4). The point in the stress-strain curve where the stress starts to drop is termed the first crack point, and the stress and strain corresponding to it are defined as first crack stress *σ_tc_* and first crack strain *ε_tc_*, respectively. The maximum tensile stress in the curve is defined as peak stress *σ_tp_*, and the strain corresponding to it is defined as peak strain *ε_tp_*. The maximum strain is defined as ultimate strain *ε_tu_*. The area under the whole load-deformation curve is defined as tensile fracture energy *G_t_*.

(2) $$\sigma =\frac{P}{A}$$

(3) $$\varepsilon_{l}=\frac{l_{l}}{L_{l0}}$$

(4) $$\varepsilon_{t}=\frac{l_{t}}{L_{t0}},$$

where *σ* is the tensile stress, *ε_l_* and *ε_t_* is the longitudinal and transverse strain, P is the tensile load, A is the cross-section area in the middle of specimen (40 mm × 40 mm), *l_l_* and *l_t_* is the longitudinal and transverse deformation, *L_l0_* and *L_t0_* is the gauge length of longitudinal (50 mm) and transverse (25 mm).

#### 2.3.4. Microstructure Analysis

The mineral phases of RFA and NFA were detected using X-ray diffraction (XRD) (PANalytical X’Pert3 Powder, Netherlands, Cu-Kα, voltage 40 kV, current 40 mA, scan speed 0.04 s/step, step size 0.013°). The defects of two kinds of high ductility cementitious composites suffering from loading were detected using X-ray Computed tomography (CT) (Xradia 410 Versa, ZEISS, Germany). The working voltage and power of the X-ray tube were 140 kV and 10 W, respectively, and the ORS Visual software was used to analyze the test results. The interface between aggregate and cement paste, fiber and matrix after tensile failure of R-HDCC and N-HDCC were obtained by using a scanning electron microscope (SEM) (EVO HD15, ZEISS, voltage 10 kV).

## 3. Results and Discussion

### 3.1. Properties of RFA and NFA

**Figure 5:**
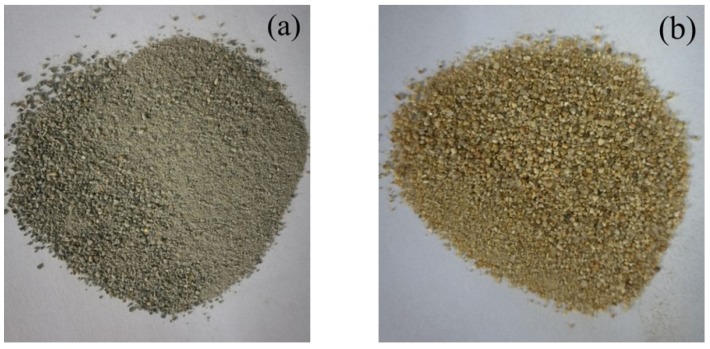
Photos of (**a**) RFA and (**b**) natural fine aggregate (NFA).

**Figure 6:**
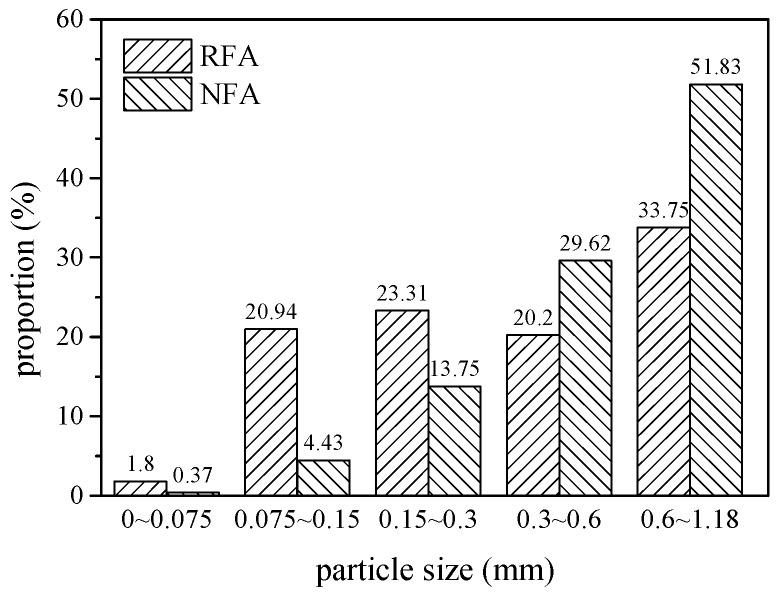
Particle size distribution of RFA and NFA.

Figure 5 shows the photos of the two kinds of fine aggregate. As can be seen, the surface of RFA is angular and rough, while that of NFA is comparatively smooth. The particle of RFA is finer than NFA generally, which is proved by the tested particle size distribution, as shown in Figure 6. RFA has scattered particle sizes while NFA particles are mainly concentrated at 0.3~1.18 mm. The particles’ proportions of 0.075~0.15 mm and 0.15~0.3 mm of RFA are 20.94% and 23.31%, which are 372.69% and 69.53% higher than those of NFA, respectively. Furthermore, RFA contains 4.86 times as much concrete crush dust (particles < 0.075 mm) as NFA.

The main physical properties of RFA and NFA, tested according to Chinese Standard GB/T 14864-2011 and GB/T 25176-2010, are listed in Table 4. As can be seen, the bulky density of RFA is 10.34% smaller than that of NFA. The water absorption and crushing index of RFA is high, up to 6.72% and 18.3%, which is 5.69 and 1.43 times of those of NFA, respectively.

**Figure 7:**
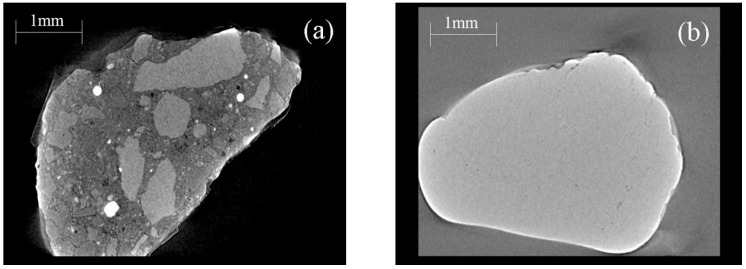
Computed tomography (CT) images of fine aggregate: (**a**) RFA and (**b**) NFA.

Figure 7 shows the CT images. It is found that RFA is a heterogeneous material consisting of NFA, natural coarse aggregate and original cement paste (Figure 7a), while NFA is homogeneous (Figure 7b), which is the main reason for the difference in physical properties between RFA and NFA.

**Figure 8:**
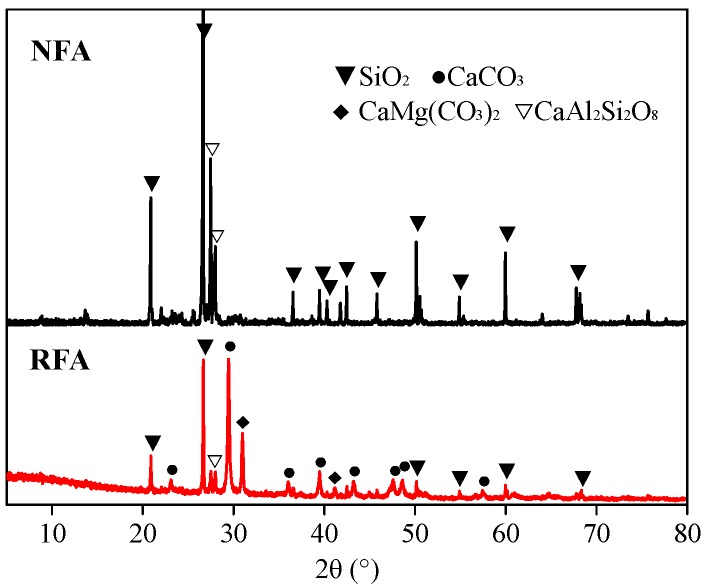
X-ray diffraction (XRD) patterns of NFA and RFA.

Figure 8 shows the XRD patterns of the two kinds of fine aggregate. RFA primarily consists of SiO_2_, CaCO_3_ and CaMg(CO_3_)_2_, while NFA is mainly composed of SiO_2_. The CaCO_3_ and CaMg(CO_3_)_2_ are introduced by the natural coarse aggregate existing in RFA, corresponding well with the CT images (Figure 7). Meanwhile, there is a small amount of CaAl_2_Si_2_O_8_ in both aggregates.

### 3.2. Compressive and Flexural Strength

**Figure 9:**
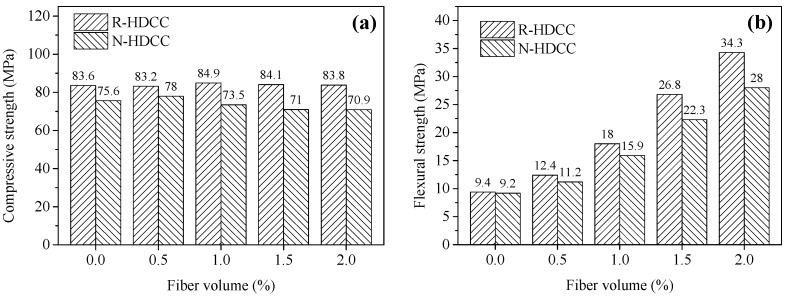
Comparison of strength between R-HDCC and N-HDCC: (**a**) Compressive and (**b**) Flexural.

The average compressive and flexural strengths of R-HDCC and N-HDCC with different fiber volume fractions (*V_f_*) are presented in Figure 9. With the increasing *V_f_*, both R-HDCC and N-HDCC show an indistinct fluctuation in compressive strength and a significant increase in flexural strength. Moreover, for specimens with 0%, 0.5%, 1.0%, 1.5% and 2.0% fiber, the compressive strengths of R-HDCC are 10.58%, 6.67%, 15.51%, 18.45% and 18.19% higher than those of N-HDCC, respectively, illustrating the higher matrix strength of R-HDCC. This can be attributed to the denser matrix of R-HDCC caused by the following four factors: First, a lot of old cement paste crumbs and concrete crush dusts existing in RFA have a certain activity that can promote hydration; Second, the concrete crush dust can form a good particle gradation with cement, fly ash and RFA, and fill in the interfacial transition zones and the gaps between the cement hydration products, as mentioned by Lederer et al. [23]; Third, RFA possesses a larger proportion of finer particles and rougher surfaces; Fourth, due to the higher water absorption rate of RFA as listed in Table 4, the water on the surface of RFA will be absorbed during the hydration reaction because of the unbalanced pressure inside and outside the RFA. The thickness of water film between the RFA and cement paste is therefore reduced, resulting in a tighter RFA/cement paste interface.

For specimens with 0%, 0.5%, 1.0%, 1.5% and 2.0% fiber, the flexural strengths of R-HDCC are 2.17%, 10.71%, 13.21%, 20.18% and 22.5% higher than those of N-HDCC, respectively. In addition, as *V_f_* increases from 0% to 2%, the flexural strength of R-HDCC increases by 264.89%, while that of N-HDCC only increases by 204.35%, demonstrating the higher enhancement of fibers in R-HDCC. This should be attributed to the higher bond between fiber and matrix of R-HDCC, which is caused by the denser matrix as mentioned above.

### 3.3. Bending Stress-Deflection Curves

**Figure 10:**
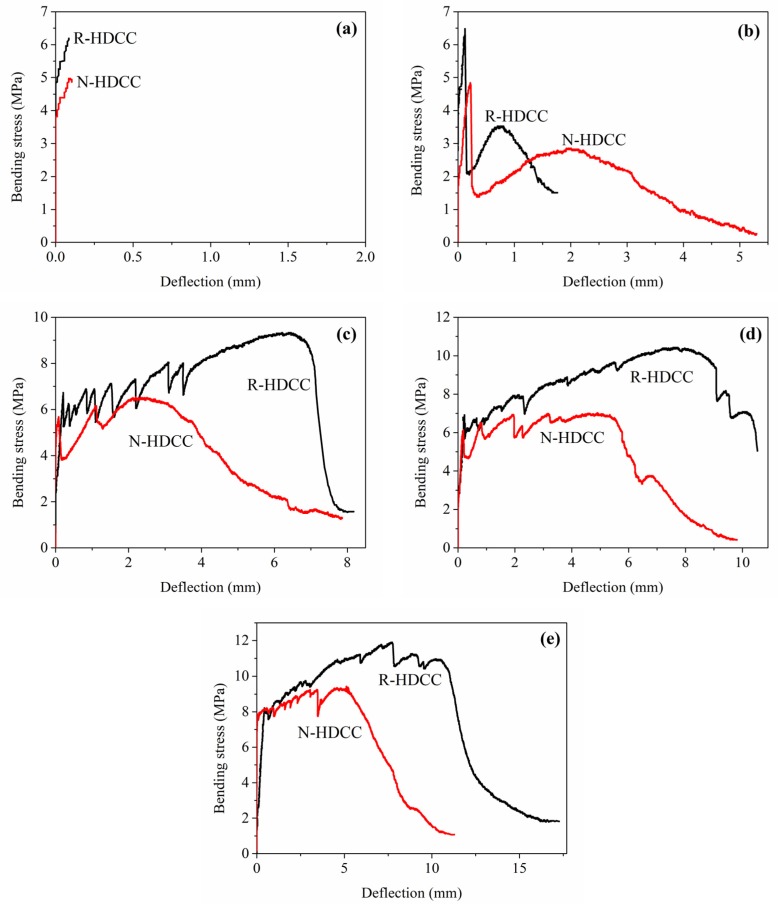
Bending stress versus deflection of R-HDCC and N-HDCC: (**a**) *V_f_* = 0, (**b**) *V_f_* = 0.5%, (**c**) *V_f_* = 1.0%, (**d**) *V_f_* = 1.5% and (**e**) *V_f_* = 2.0%.

**Figure 11:**
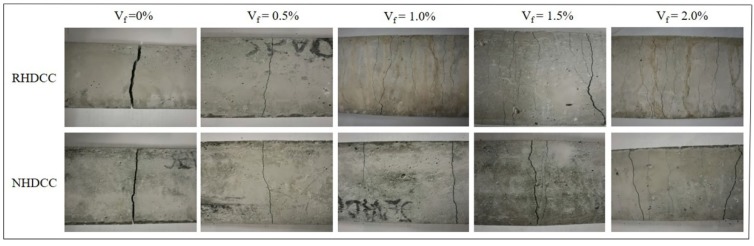
Bottom surface of specimens after bending failure.

The four-point bending stress-deflection curves of R-HDCC and N-HDCC with *V_f_* varying from 0% to 2.0% are shown in Figure 10a–e, and the bottom surfaces of tested specimens are shown in Figure 11. The performance parameters calculated according to curves are presented in Table 5.

It can be observed that specimens without fiber presented a brittle failure. As shown in Figure 10a, the bending stress-deflection curves of two types of HDCC exhibit a similar shape that tends to be linearly elastic until crack occurs and then the specimen suddenly fractures, as shown in Figure 11. Finally, as compared with N-HDCC (25N00), R-HDCC (25R00) produces a 27.1% increase in bending stress and a 10% decrease in deflection, as presented in Table 5. The fracture energy of R-HDCC is almost no different to that of N-HDCC in this case.

The specimens with 0.5% fibers failed in the ductile mode and the bending stress-deflection curves become fatter (Figure 10b), which reflects a higher ductility compared with the plain mortar. The deflection hardening process does not appear because the maximum fiber bridging stress at this dosage is smaller than the cracking strength of the matrix. The fibers that bridge across cracks are ruptured and the onset of multiple cracking is arrested as depicted by the first cracking strength criterion [39]. Both R-HDCC and N-HDCC present increasing performance parameters with the addition of fiber, as shown in Table 5. Moreover, when *V_f_* = 0.5%, the *σ_bc_* and *σ_bp_* of R-HDCC (25R05) are 6.25 MPa and 6.47 MPa, which is 30.75% and 33.68% higher than those of N-HDCC (25R05), respectively. On the contrary, the *δ_bc_*, *δ_bp_*, *δ_bu_* and *G_b_* of R-HDCC are 47.62%, 45.45%, 66.79% and 48.18% lower than those of N-HDCC, respectively. This should be attributed to the higher bond between fiber and matrix as explained in Section 3.2., which makes fibers in R-HDCC easier to rupture. Therefore, the reinforced effects of fibers on the ductility and energy absorption ability of R-HDCC has not been well exploited.

For specimens with *V_f_* ≥ 1.0%, R-HDCC failed with multiple cracking and exhibits an observable deflection hardening; however, this phenomenon of N-HDCC is less remarkable, as shown in Figure 10c–e and Figure 11. Most of the fibers are pulled out and the whole failure process develops as follows: the stress increases in proportion to the deflection until the first crack appears, and continues to increase up to the first peak point, then it drops slightly and immediately rises again through the fiber bridging effect. Once the stress exceeds the matrix cracking strength, new cracks will appear and the stress will decrease slightly again. This process is repeated until the microcracks are saturated. Subsequently, the number of cracks no longer increases, but the width continues to increase. Finally, a localized crack opening occurs at one of the weak sections and the stress decreases continuously, causing the failure of the specimen.

R-HDCC presents many advantages compared with N-HDCC when *V_f_* ≥ 1.0%. First, as shown in Figure 10c–e, the height and plumpness of the whole curves of R-HDCC are much higher than those of N-HDCC, which shows that R-HDCC has higher bending strength and ductility. Second, as presented in Table 5, all performance parameters of R-HDCC are much higher than those of N-HDCC. The *δ_bp_* of R-HDCC (25R10), especially, is 182.73% higher than that of N-HDCC (25N10) when *V_f_* = 1.0%, and the *G_b_* of R-HDCC (25R20) is 95.22% higher than that of N-HDCC (25N20) when *V_f_* = 2.0%. The higher bond between fiber and matrix interface leads to higher fiber bridging stress, and thus the fiber deformation is larger. Meanwhile, in the condition of strain hardening, the higher bond between fiber and matrix interface can enhance the development of multiple cracking. The lower elastic module of RFA also contributes to the larger deformation of R-HDCC. In addition, it needs less detour for cracks to propagate due to the finer particle size of RFA, as described in Figure 6, which greatly promotes the multiple cracking according to the crack trapping mechanism [23]. As a result, R-HDCC achieves a higher peak load, larger peak deflection and better energy absorption ability than N-HDCC.

### 3.4. Axial Tensile Stress-Strain Curves

**Figure 12:**
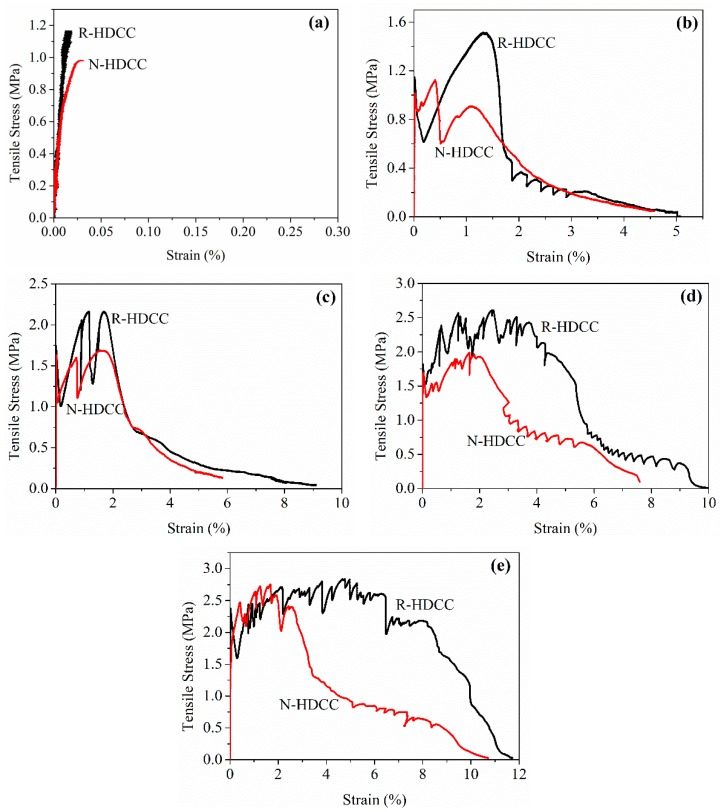
Stress versus strain of R-HDCC and N-HDCC: (**a**) *V_f_* = 0, (**b**) *V_f_* = 0.5%, (**c**) *V_f_* = 1.0%, (**d**) *V_f_* = 1.5% and (**e**) *V_f_* = 2.0%.

**Figure 13:**
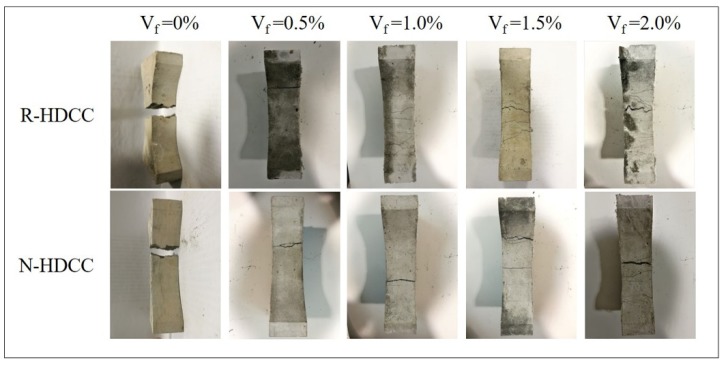
Dog-bone specimens after axial tensile test.

**Figure 14:**
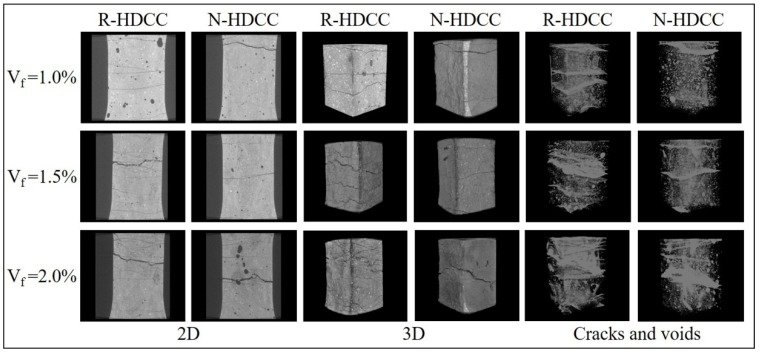
CT images on specimens after axial tensile test.

The tensile stress-strain curves of R-HDCC and N-HDCC with *V_f_* varying from 0% to 2.0% are presented in Figure 12a–e, and the failure modes of specimens are displayed in Figure 13. After the tensile test, the defects including cracks and voids in the gauge length of specimens with *V_f_* = 1.0%~2.0% were scanned by CT, as shown in Figure 14. The tensile properties calculated according to stress-strain curve are listed in Table 6.

For specimens without fibers, the tensile stress increases with the increase in strain until the occurrence of the crack (Figure 12a), and then it was broken into two halves (Figure 13). The *σ_tc_* and *E_t_* of R-HDCC (45R00) are 17.35% and 80.12% higher whereas the *ε_tc_* and *G_t_* of R-HDCC are 33.33% and 32.11% lower than those of N-HDCC (45N00), respectively, as show in Table 6.

For specimens with 0.5% and 1.0% fiber, both R-HDCC and N-HDCC failed in ductile mode and show a similar stress-strain curve shape that stress exhibits a few fluctuations and then decreases continuously as the crack opening localizes, as presented in Figure 12b,c. For specimens with 1.5% and 2.0% fibers, R-HDCC exhibits an evident strain hardening and failed with apparent multiple cracking. However, N-HDCC only exhibited some fluctuations in curve and several cracks around the major crack, as shown in Figure 12d,e and Figure 13. These phenomena can be demonstrated more intuitive and powerfully by the CT images shown in Figure 14. On the other hand, the void size of R-HDCC is smaller and is distributed in a narrow range compared to that of N-HDCC, which may promote the development of multiple cracking. The whole failure mechanism of the tensile test is similar to that of bending. The results listed in Table 6 indicate that the *σ_tp_*, *ε_tp_*, *ε_tu_*, *E_t_* and *G_t_* of R-HDCC increases continuously by 88.08%, 260.60%, 130.91%, 58.74% and 824.84% with *V_f_* increasing from 0.5% to 2.0%, showing the significant enhancement of the fiber. Moreover, all the tensile stress parameters of R-HDCC are higher than those of N-HDCC with corresponding *V_f_*. The *σ_tc_* of R-HDCC (45R20), especially, is 51.59% higher than that of N-HDCC (45N20) when *V_f_* = 2.0%, and the *σ_tp_* of R-HDCC (45R15) is 34.82% higher than that of N-HDCC (45N15) when *V_f_* = 1.5%. Similarly, the *E_t_* of R-HDCC is generally higher than that of N-HDCC under tensile load. Especially for specimens with 2.0% fiber, the *E_t_* of R-HDCC is 20.35 GPa, which is 56.9% higher than that of N-HDCC. Furthermore, R-HDCC with 2.0% fiber exhibits a superior tensile behavior with *ε_tp_* up to 4.76% and *ε_tu_* up to 11.73%, which are 2.83 and 1.09 times of those of N-HDCC, respectively. R-HDCC also has a better energy absorption ability with a much higher *G_t_* than N-HDCC, except for specimens without fiber. The *ν_t_* is in the range of 0.1~0.3 without regular change. It can be concluded that R-HDCC possesses better load carrying capacity, higher ductility and greater energy absorption ability than N-HDCC under axial tensile load. This should be attributed to the same reasons as explained above for bending.

**Figure 15:**
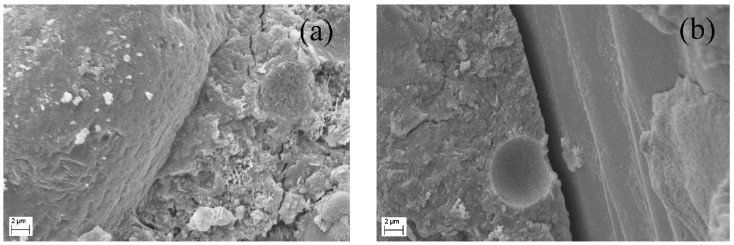
Scanning electron microscopy (SEM) images of interfacial transition zone between aggregate and cement paste: (**a**) RFA and (**b**) NFA.

**Figure 16:**
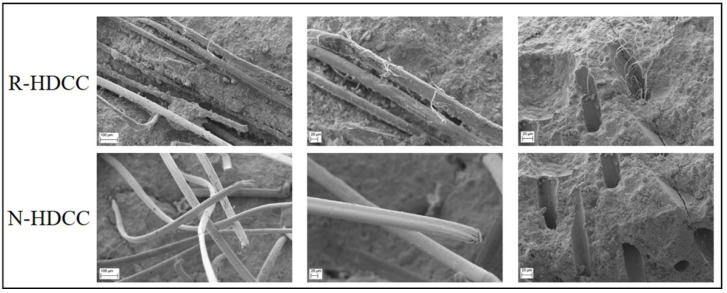
SEM images of fiber/matrix interface after tensile test.

In order to confirm the inferences in mechanical analysis, the interfacial transition zones between aggregate and cement paste, fiber and matrix in R-HDCC and N-HDCC were investigated through SEM after tensile test. As shown in Figure 15, there is nearly no space between RFA and cement paste, while there is an obvious gap between NFA and cement paste, which indicates a tighter RFA/cement paste interface of R-HDCC, corresponding well with the explanation for compressive strength. Moreover, as shown in Figure 16, after being pulled out from R-HDCC, the fibers presented a rough surface with a large amount of hydrated product. Meanwhile, the fibers were so seriously damaged that the surface filaments were stripped and remained in the holes during pull-out, whereas the surfaces of the fibers pulled out from N-HDCC were relatively smooth with few abrasions. This shows that the bond between fibers and matrix in R-HDCC is higher than that in N-HDCC, which shows an agreement with the inferences in bending and tension. All the test results effectively demonstrate that R-HDCC exhibits better mechanical properties than N-HDCC.

## 4. Prediction of Mechanical Properties

**Figure 17:**
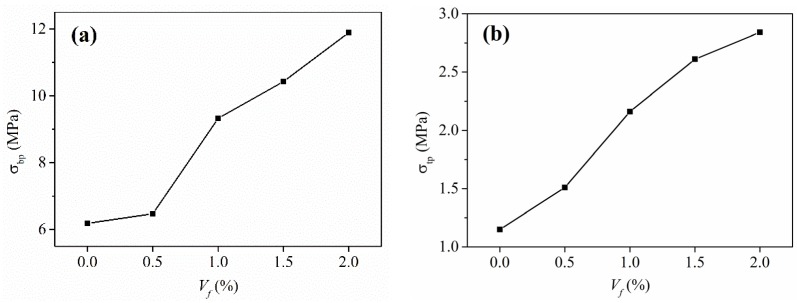
Effect of fiber volume fraction: (**a**) bending peak stress and (**b**) tensile peak stress.

It can be seen in Figure 9a that the fiber content *V_f_* has little impact on the compressive strength *f_c_* of R-HDCC. However, the bending peak stress *σ_bp_* and tensile peak stress *σ_tp_* of R-HDCC apparently linearly increase with the increase of *V_f_*, as shown in Figure 17. The relations between various peak strengths and *V_f_* were treated by the normalization of the peak stress to eliminate the influence of matrix strength. Then, the prediction models were established by linear regression.

### 4.1. Bending Peak Strength

**Figure 18:**
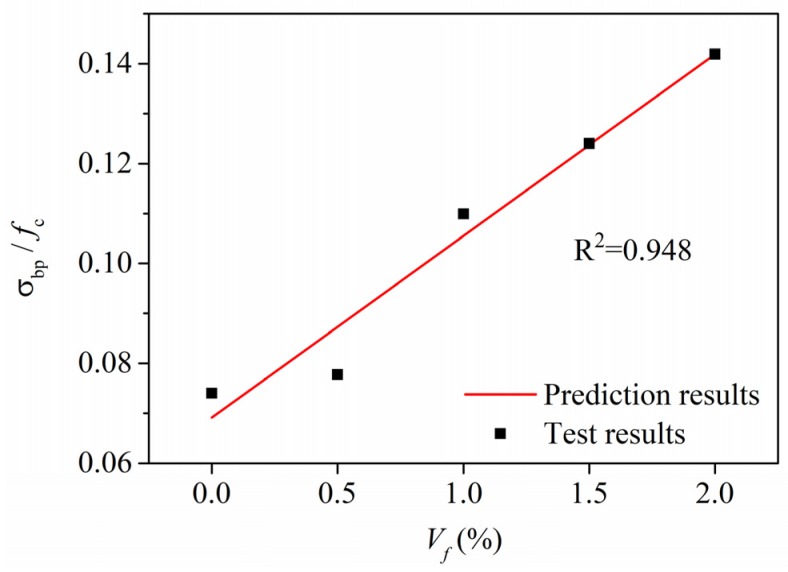
Relationship between *σ_bp_/f_c_* and *V_f_*.

From the experimental results shown in Figure 18, the value of *σ_bp_*/*f*_c_ of R-HDCC linearly increases with the increase in *V_f_*, thus the relationship between *σ_bp_*/*f*_c_ and *V_f_* can be modelled by linear fitting and expressed as Equation (5). The solid line of Equation (5) in Figure 18 shows good agreement with the test results with R^2^ = 0.948.

*σ_bp_/f_c_*= 0.036 *V_f_* + 0.069 (5)

### 4.2. Tensile Peak Strength

**Figure 19:**
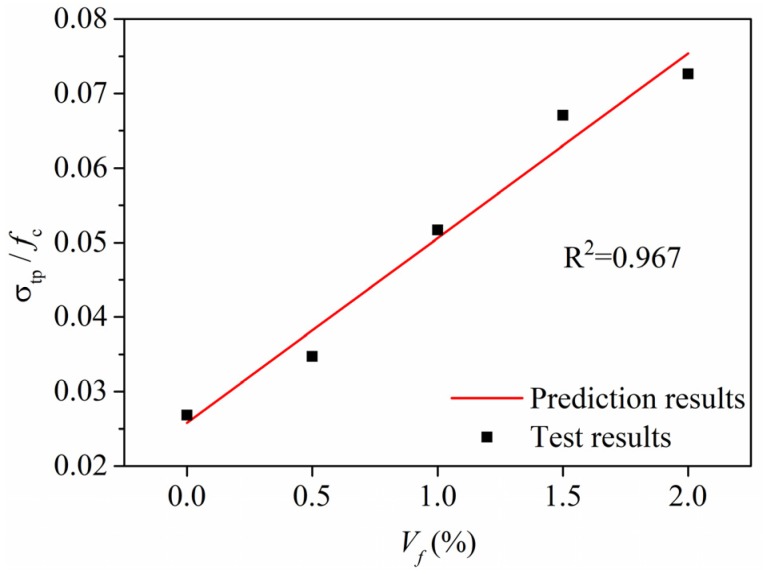
Relationship between *σ_tp_/f_c_* and *V_f_*.

The value of *σ_tp_/f_c_* of R-HDCC also linearly increases with the increase of *V_f_* as shown in Figure 19. The relationship between *σ_tp_/f_c_* and *V_f_* is modelled by linear fitting and expressed as Equation (6). The solid line of Equation (6) in Figure 19 shows good agreement with the test results with R^2^ = 0.967.

*σ_tp_/f_c_* = 0.025 *V_f_* + 0.026 (6)

**Figure 20:**
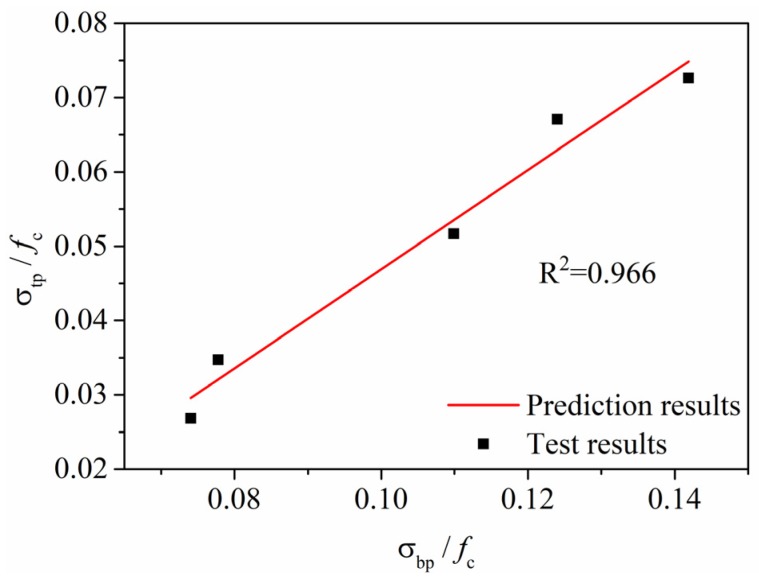
Relationship between *σ_bp_/f_c_* and *σ_tp_/f_c_*_._

In addition, the values of *σ_bp_/f_c_* also show a good linear relationship with *σ_tp_/f_c_*, as shown in Figure 20 and modelled as Equation (7). The solid line of Equation (7) in Figure 20 shows good agreement with the test results with R^2^ = 0.966.

*σ_tp_/f_c_* = 0.668 *σ_bp_/f_c_* ‒ 0.02 (7)

## 5. Conclusions

RFA with original particle size distribution was used to fully replace NFA to prepare HDCC in this study. The features of RFA and NFA were tested in detail. The mechanical properties and interface microstructure of HDCC prepared with RFA and NFA were investigated and compared, and the following conclusions can be drawn:

(1) RFA with original particle size distribution contains 4.86 times as much concrete crush dust as natural fine aggregate. The dust supplies a certain activity and a good filling effect in matrix. Coupled with the higher water absorption rate of RFA, HDCC with RFA forms a denser matrix. Thus, HDCC with RFA exhibits a higher compressive strength than HDCC with NFA.
(2) Because of the denser matrix, HDCC with RFA has a higher bond between fiber and matrix than HDCC with NFA. This can be proved by the scanning electron microscope observations that fibers pulled out from HDCC with RFA were seriously damaged while fibers pulled out from HDCC with NFA were only slightly abraded. As a result, HDCC with RFA exhibits higher bending and tensile strength than HDCC with NFA.
(3) The higher bond between fiber and matrix of HDCC with RFA and the finer particle sizes of RFA can greatly promote the development of multiple cracking. Thus, HDCC with RFA presents more remarkable stain hardening and exhibits 182.73% higher peak deflection in the bending and 183.33% higher peak strain in tension than HDCC with NFA.
(4) The values of σ_bp_/*f*_c_ and σ_tp_/*f*_c_ of HDCC with RFA linearly increase with the increase of *V_f_*, and the relationships are modelled by a linear equation, respectively. Additionally, there is also a good linear relationship between the values of σ_bp_/*f*_c_ and σ_tp_/*f*_c_ of HDCC with RFA.
(5) HDCC with RFA exhibits better mechanical properties than HDCC with NFA. Therefore, the application of RFA in the preparation of HDCC can obtain significant social and economic benefits.

## Figures and Tables

**Table 1:** Chemical composition of cement and fly ash (wt%).

Chemical Composition	Cement (%)	Fly Ash (%)
CaO	63.42	4.2
SiO_2_	18.77	54.45
Al_2_O_3_	4.85	28.37
MgO	4.17	0.85
SO_3_	3.53	0.85
Fe_2_O_3_	3.15	6.03
K_2_O	1.2	2.29
T_i_O_2_	0.255	1.25
Na_2_O	0.243	0.94
loss of ignition	3.08	3.17

**Table 2:** Properties of polyvinyl alcohol (PVA) fiber.

Tensile Strength (MPa)	Modulus of Elasticity (GPa)	Elongation (%)	Length (mm)	Diameter (μm)	Density (g/m^3^)
≥1250	>30	≥6	12	40	1.3

**Table 3:** Mix proportions of raw materials.

Group	Parameters					Mix Proportion (kg/m^3^)					
	*W/B*	*V_f_* (%)	*R/B*	*V_FA_* (%)	Aggregate Type	Cement	Fly Ash	Fine Aggregate	Fiber	Water	Water Reducer
25R00	0.25	0	0.4	30	RFA	831.6	356.4	475.2	0	297	12.1
25R05	0.25	0.5	0.4	30	RFA	831.6	356.4	475.2	6.5	297	12.1
25R10	0.25	1	0.4	30	RFA	831.6	356.4	475.2	13	297	12.1
25R15	0.25	1.5	0.4	30	RFA	831.6	356.4	475.2	19.5	297	12.1
25R20	0.25	2	0.4	30	RFA	831.6	356.4	475.2	26	297	12.1
25N00	0.25	0	0.4	30	NFA	831.6	356.4	475.2	0	297	12.1
25N05	0.25	0.5	0.4	30	NFA	831.6	356.4	475.2	6.5	297	12.1
25N10	0.25	1	0.4	30	NFA	831.6	356.4	475.2	13	297	12.1
25N15	0.25	1.5	0.4	30	NFA	831.6	356.4	475.2	19.5	297	12.1
25N20	0.25	2	0.4	30	NFA	831.6	356.4	475.2	26	297	12.1
45R00	0.45	0	0.4	30	RFA	626.9	268.6	358.2	0	403	0
45R05	0.45	0.5	0.4	30	RFA	626.9	268.6	358.2	6.5	403	0
45R10	0.45	1	0.4	30	RFA	626.9	268.6	358.2	13	403	0
45R15	0.45	1.5	0.4	30	RFA	626.9	268.6	358.2	19.5	403	0
45R20	0.45	2	0.4	30	RFA	626.9	268.6	358.2	26	403	0
45N00	0.45	0	0.4	30	NFA	626.9	268.6	358.2	0	403	0
45N05	0.45	0.5	0.4	30	NFA	626.9	268.6	358.2	6.5	403	0
45N10	0.45	1	0.4	30	NFA	626.9	268.6	358.2	13	403	0
45N15	0.45	1.5	0.4	30	NFA	626.9	268.6	358.2	19.5	403	0
45N20	0.45	2	0.4	30	NFA	626.9	268.6	358.2	26	403	0

Note: *W/B* is water-binder ratio, *V_f_* is fiber volume fraction, *R/B* is RFA-binder ratio, *V*_FA_ is the proportion of fly ash replacing cement.

**Table 4:** Physical properties of RFA and NFA.

Aggregate Type	Apparent Density (kg/m^3^)	Bulky Density (kg/m^3^)	Water Absorption (%)	Crushing Index (%)
RFA	2536	1309	6.72	18.3
NFA	2593	1460	1.18	12.8

**Table 5:** Bending properties.

Group	*σ_bc_* (MPa)	*δ_bc_* (mm)	*σ_bp_* (MPa)	*δ_bp_* (mm)	*δ_bu_* (mm)	*G_b_*
25R00	6.19	0.09	/	/	/	35.20
25N00	4.87	0.10	/	/	/	35.01
25R05	6.25	0.11	6.47	0.12	1.76	373.17
25N05	4.78	0.21	4.84	0.22	5.30	720.10
25R10	6.57	0.20	9.33	6.22	8.17	4308.87
25N10	5.58	0.08	6.54	2.20	7.86	2562.66
25R15	6.78	0.23	10.43	7.58	10.53	6738.25
25N15	6.06	0.18	7.02	4.90	9.81	3451.62
25R20	8.11	0.42	11.89	7.67	17.26	10090.39
25N20	7.97	0.21	9.41	5.12	11.28	5403.01

**Table 6:** Tensile properties.

Group	*σ_tc_* (MPa)	*ε_tc_* (%)	*σ_tp_* (MPa)	*ε_tp_* (%)	*ε_tu_* (%)	*E_t_* (GPa)	*G_t_*	*ν_t_*
45R00	1.15	0.02	\	\	\	11.49	12.54	0.18
45N00	0.98	0.03	\	\	\	6.49	18.47	0.12
45R05	1.14	0.01	1.51	1.32	5.08	12.82	2033.34	0.16
45N05	1.02	0.02	1.12	0.41	4.58	7.76	1634.00	0.30
45R10	1.75	0.01	2.16	1.69	9.10	13.29	4796.38	0.10
45N10	1.63	0.01	1.69	1.54	5.83	10.66	3872.03	0.14
45R15	1.82	0.02	2.61	2.48	10.02	16.01	11002.04	0.21
45N15	1.70	0.04	1.99	1.64	7.60	11.38	6263.50	0.13
45R20	2.38	0.02	2.84	4.76	11.73	20.35	18805.06	0.13
45N20	1.57	0.05	2.75	1.68	10.72	12.97	10143.70	0.15

## Abbreviation

RFA	recycled fine aggregate;
NFA	natural fine aggregate;
HDCC	high ductility cementitious composite;
R-HDCC	high ductility cementitious composite prepared with recycled fine aggregate;
N-HDCC	high ductility cementitious composite prepared with natural fine aggregate.
